# A Thesis on Vesico-Vaginal-Fistula

**Published:** 1857-11

**Authors:** Camillus Hilliard

**Affiliations:** Montgomery, Ala.


ATLANTA

Medical and Surgical Journal.

Vol. III.] NOVEMBER, 1857. [No. III.

### ORIGINAL COMMUNICATIONS.

### ARTICLE I.

*A Thesis on Vesico-Vaginal-Fistula. Presented to the Faculty of the Atlanta Medical College for the Degree of Doctor of Medicine.* By Camillus Hilliard, of Montgomery, Ala.

*Honored Sirs:* It has been aptly remarked, that this is an age of progress. In no department of science is this more apparent than in that of medicine. In the last half century, such gigantic strides have been made in this science, that contemplating what has been accomplished, we are astounded. Looking back to Hippocrates, the "Father of Medicine," we see this glorious and noble art, beginning as doth a rill in the far distant mountains, flowing on through succeeding centuries, it receives accessions from varied sources such as Italy, Persia, Arabia, etc. Now and then, its clear stream muddied by some false theory, but again becoming clear under the searching eye of science—damned up for awhile by the cursed dogmatism of Benedictine-Monks and like sects, but as the stream to which I have compared it, swelling and heaving until it removed the barriers opposed to it, always sustaining itself by its dignity and humanity. Until now instead of increasing by centuries, almost yearly new light is thrown upon it, and its waters begin to glisten under the halo science has cast over it.

You will not deem it strange, respected sirs, that I have made these general remarks, when you remember that until within the last five years the prognosis of Vesico-Vaginal-Fistula (the subject of my Thesis,) was universally considered unfavorable.

Reading the works of the older authors upon the subjects connected with Surgery, you would scarcely dream that a disease so painful and pitiable as this is in its consequences existed. One which so disfigures "the last, best gift of God to man," that instead of the feelings of unmitigated delight and respect with which we usually behold them, we feel only disgust and loathing, mixed in the humane person with a feeling, perhaps, of pity.

The older authors considering this affection as one beyond the reach of medical art or surgical skill, thought it a mere waste of time and display of ignorance, to say anything about it. Even in reviewing the written opinions of more modern Surgeons, we find that it is but recently, that any advance has been made, either in the treatment or proper understanding of it. It has been described, and modes of treatment proposed, but not much notice was taken of either. Within the last quarter of a century, however, several eminent Surgeons having turned their attention to it, it is with pleasure I state, there is scarcely any disease in the whole range of Surgery, more clearly understood or more easily treated.

I will now proceed immediately to the consideration of the subject.

*Anatomy.*—Between the bladder and anterior wall of the vagina, there is a small quantity of areolar tissue. The union of the two is called the Vesico-Vaginal-Septum, which is composed of muscular and erectile tissue, covered on both sides by mucus membrane. The shape of this Septum resembles somewhat a heart on playing cards, its base presenting upwards, the cervix uteri occupying the notch, the apex pointing downwards to the opening of the urethra.

The opening constituting the fistula may occur at any point, but is usually near the centre of the Septum. In the several cases I have had the fortune to see operated upon, the fistules were of various sizes and number, some patients having one, others two. The form is generally oval, though it may of course be any. The size varies from an ⅛ of an inch to 3 or 4; in fact, the whole Septum may slough away and allow the fundus of the bladder to protrude through, presenting the appearance of a large fleshy tumor.

The presence of more than one fistula, is a circumstance of considerable importance in reference to the chances of successful treatment. The reason of this is, that if there be more than one, dribbling of the urine is more likely to occur, and by its action, prevent the process of reparation.

*The Causes.*—These are various, such as impaction of the child's head during labor. The soft parts of the mother being grasped as in a vice, between the presenting part of the child and the bony walls of the pelvis, arresting circulation and causing the parts compressed to slough away. The misuse of forceps. Lithotomy performed through the vagina. Disease of cervix uteri, etc.

*Diagnosis.*—This is easy, the symptoms being few, but well marked. When it has followed tedious labor, retention of urine is first noticed, it being necessary to use the catheter for the relief of the bladder. It may continue for several days, from seven to twelve, and then be converted into complete incontinence, the sloughs having fallen, and a continued involuntary stillicidium being established. A probe being passed into the urethra will come into contact, through the fistula, with a finger passed into the vagina. A specular examination will prove with certainty the existence, shape, size, etc., of the fistula.

*Treatment.*—This can be embraced under two general heads, viz: Suture and Cauterization. Cauterization is divided into chemical and actual cautery. The former is applicable only in cases where the opening is small; it heals by granulation. The actual cautery cures by contraction of the cicatrix. It should be applied at a white heat for an instant around the edges of the opening. Some authors state that there should be from four to five weeks between each application; others, from two to three months, so as to allow the cicatrix full time to contract. From all that I can gather upon the subject, I do not like this mode of treatment, it being in my opinion, generally unsuccessful, the opening contracting to one or two lines in diameter, and then resisting all further attempts to close it. Liston, however, sanctions it, and he stands deservedly high in the ranks of Surgery.

*The Suture.*—There are numerous modifications of this, as the twisted, the plastic, the clamp, and the button suture.

The Suture had its origin in Germany. The name of the Physician who proposed its use has escaped my memory. It then fell into disuse, but was snatched from oblivion in the beginning of the present century. Since that time the suture has been employed in Germany in different forms, and in the hands of some has proven very successful. One of the most succcessful of their Surgeons joined puncture of the bladder with his operations, so that its contents might not retard the progress of recovery by their irritating influence on the raw edges.

Velpean condemns the Suture in the heartiest language, and experience has proved unjustly. He winds up his remarks by saying that such a mode of treatment, appears to be calculated to have no other result than to cause unnecessary suffering to the patient.

In England, very little has been done to relieve this malady. The Suture has been employed there to some extent, but with variable success.

Coming now to the United States, I feel very much like devoting the rest of my pages to a eulogy on our glorious country. Knowing this would not be considered exactly "the thing," in an article of this kind, I will try to suppress my patriotic feelings, and content myself by saying, that in this, as in everything else, our countrymen have shown themselves the equals, if not the superiors, of phlegmatic Germany, polite France, and sturdy England combined.

To the names of Pancoast, Hayward, Sims, Mattaeur, Bozeman and some other American surgeons the world should be grateful, for they have placed the prognosis of Vesico-vaginal

Fistula on a par with diseases of like severity. Dr. Mattauer was one of the first who operated in this country; he first pared the edges of the opening and then introduced sutures of lead wire, and brought the edges together by twisting the sutures; he clips the wires just exterior to the vulva, tightens them on the fourth day, and removes them on the tenth.

The only peculiarity in Dr. Haywards operation is, that he introduces a whalebone bougie, and with this as a lever and the os pubis as a fulcrum he brings down the fistula so as to be more easily seen and operated upon. He allowed the sutures to come away by ulceration.

Dr. PANCOAST's operation differs from others in the manner in which he pares the edges, he slits the posterior and shapes the anterior in the form of a wedge, he thus approximates four raw surfaces by his plastic suture.

The different surgeons employ different methods of getting at the parts. Drs. Sims & Bozeman use the same position, which I will describe. The patient is placed upon a table, (covered with folded blankets, for the purpose of making it softer;) upon all fours with her arms folded and head lowered upon them. The table is drawn close to a window so that the rays of sun which come through can be reflected by means of a mirror into the vagina; as it is necessary to have all the "light on the subject" possible, so that every nook and corner of this "terra incognita" may be clearly exhibited to the searcher after openings. There should be three assistants, one to introduce and retain the speculum "in situ," one to attend the sponges and the other to separate the nates of the patient, so that the operator may have a clear field for action, as this is always desirable. A very bright day should be selected for the purpose. The patient should not be disturbed until everything is in readiness to proceed immediately, as the operation is a very fatiguing one. The day before operating the bowels should be thoroughly purged with castor oil, and then on the day of the operation locked up, (so as to prevent all straining of the parts,) by opium. I must here, although it is somewhat out of place, recommend the table proposed by Dr. P. M. Kollock, the talented professor of obstetrics in the Savannah Medical College, for a description of which I respectfully refer you to his article in the May and June numbers of the Augusta Medical Journal. It greatly adds to the comfort of the patient and convenience of the surgeon.

Dr. Sims claims that he has originated, 1st. A method by which the vagina can be thoroughly explored. 2nd. That he has introduced a new suture apparatus which lies imbedded in the tissues for an indefinite time without danger of cutting its way out as do silk ligatures. 3d. That he has invented a self-retaining catheter, which may be worn with great comfort to the patient during the whole course of treatment.

His method of operating is as follows: Having placed the patient upon the table, in the manner described, introduced the speculum and thrown light into the vagina, he raises the mucous membrane surrounding the fistula by the aid of a delicate tenaculum inserted into a handle some six inches in length, then with a small knife the same length he pares of this membrane around the orifice for a $\frac{1}{4}$ or $\frac{1}{3}$ of an inch. After this he takes a spear-pointed needle, fixed in a long shaft, and armed with silk thread and introduces it at the distance of $\frac{1}{2}$ an inch from the pared edges, the suture only penetrates the mucous membrane and does not enter the bladder and is brought out at the same distance from the upper or posterior lid of the fistula. The needle is withdrawn, and the proximal end of the silk is attached to the end of the silver-wire, then drawing upon the distal extremity of the thread he draws the wire into place, then taking two metallic rods suited in their length to the size of the fistula he runs the distal ends of the wire through holes made in the rod for that purpose and wraps the wires around the clamp to secure them, and lodges the clamp in its place at the upper end of the opening. Another clamp of the same description is now threaded with the proximal ends of the wire and pushed into position at the lower edge of the fistula by means of a species of fork, then making traction on the upper and at the same time pushing up the lower clamp, he approximates the edges. Perforated bird shot are then run on the wires, pushed up to the lower clamp and there compressed by forceps so as to secure them. The woman is placed in bed and a catheter introduced, attention should be paid to cleansing it when necessary.

Now the objections to Dr. Sims operation, are, 1st. Notwithstanding his assertion the sutures will cut out as is shown by the experience of Drs. Bozeman, Kollock, Mattaeur and others, they certainly do not do so as readily as silk sutures, but they do cut out. This may result in the formation of other fistules, and if this does not occur, the morbid action is liable to extend to the edges of the fistula and thus prevent the healing process. 2nd. Gangrene and sloughing of the parts may occur when the clamps are applied too tightly.—3rdly. It does not prevent, if there are two openings, the urine from acting on the edges, if by any accident one of the fistules should fail to heal.

Now Dr. Bozeman's operation obviates all these difficulties, and, besides this, has the advantage, that by it any number of fistules can be operated on at the same time, whereas it is often impossible to close more than one at a time by Dr. Sims' method from the unfavorable position of the openings.

I will now describe Dr. Bozeman's operation. The first steps are very nearly similar to Dr. Sims'. The wires being lodged in their places, the ends are run through a hole in a steel rod, flattened on one side and raised into a knob on the other, at its distal end and perforated for the purpose—called "the suture adjuster"—this instrument is then run down to the edges of the fistula at every point where the wires have been lodged. A button is then taken, which may be made out of lead or silver, the former answers every purpose when hammered out to the 1-16 of an inch in thickness—it may be made of any size or shape to suit the fistula. The under surface must be concave and the edges sligtly turned up to prevent irritation of the walls of the vagina by their sharpness. The double wires are then passed through holes in the button, and this is pushed down to the edges of the fistula. A perforated shot is then run on the wires down to the button and then held fast by compression. The wires are clipped about an ⅛ of an inch above the shot and the severed ends turned over the shot. The patient is then placed in bed and Sims' catheter introduced.

Dr. Bozeman claims for his "button suture" the following advantages over the clamp suture of Dr. Sims': 1st. It is simpler in its construction and applicable to a greater number of cases. 2nd. It affords complete protection and perfect rest to the approximated edges. 3d. If there be two openings, one or both can be closed without reference to the condition of the parts. 4th. The introduction of the suture does not require the same exactness in regard to points. 5th. The independent action of each suture renders parallelism unnecessary, and thus gives the operator the privilege of introducing them in whatever direction best suits the purpose. 6th. The apparatus does not irritate, it matters not what the condition of the parts may be, so they are not in a state of ulceration or inflammation. 7th. The apparatus is required to remain seldom longer than ten days.

With due deference, I would say that any one who investigates the subject thoroughly, cannot fail to grant Dr. Bozeman all he asks. He has indeed achieved a triumph in modern surgery which the profession and the world in general should hail with gratitude.

